# Cartilage oligomeric matrix protein in patients with osteoarthritis is independently associated with metastatic disease in prostate cancer

**DOI:** 10.18632/oncotarget.27113

**Published:** 2019-07-30

**Authors:** Samuel Rosas, Ryan T. Hughes, Michael Farris, Hwajin Lee, Emory R. McTyre, Johannes F. Plate, Lihong Shi, Cynthia L. Emory, A. William Blackstock, Bethany A. Kerr, Jeffrey S. Willey

**Affiliations:** ^1^Department of Radiation Oncology, Wake Forest School of Medicine, Winston-Salem, NC, USA; ^2^Department of Orthopaedic Surgery, Wake Forest School of Medicine, Winston-Salem, NC, USA; ^3^Radiation Oncology, Greenville Health System Cancer Institute, Greenville, SC, USA; ^4^Department of Cancer Biology, Wake Forest School of Medicine, Winston-Salem, NC, USA

**Keywords:** prostate cancer, osteoarthritis, distant metastasis, arthroplasty, joint surgery

## Abstract

Metastatic prostate cancer has a 5-year survival rate of 30%. Identifying predictors of metastasis outcome could potentially reduce patient mortality. The objective of this study was to determine whether osteoarthritis had an impact on outcomes of prostate cancer including death, local recurrence and/or metastasis and to determine whether cartilage oligomeric matrix protein was involved. We performed a retrospective case-control study of patients with prostate cancer with and without the diagnosis of osteoarthritis and completed immunohistochemistry (IHC) analysis of prostate (n=20) and lymph node (n=7) surgical specimens. We evaluated death, local recurrence and metastatic disease by various IHC biomarkers including prostate specific membrane antigen (PSMA), cartilage oligomeric matrix protein (COMP), CD31, and Ki-67.

Our model identified osteoarthritis as an independent risk factor for metastatic disease (OR 5.24, 95% CI 1.49 - 18.41). Most notably, when joint arthroplasty was included in the model, osteoarthritis was no longer an independent risk factor for this outcome (p=0.071). IHC demonstrated that those with osteoarthritis, had greater expression of COMP in the prostate samples (mean 23.9% vs 5.84%, p<0.05) but not of Ki-67, CD31, or PSMA. This study identified and quantified increased metastatic disease in patients with osteoarthritis. Also, patients with osteoarthritis expressed increased COMP levels in the prostate and most likely in distant lymphatic nodes. Moreover, our findings suggest that joint arthroplasty may affect the ability of osteoarthritis to promote metastasis, which could impact treatment protocols and survival outcomes of the most common cause of cancer-related death (metastasis) in the United States.

## INTRODUCTION

Prostate cancer is the second most common cause of cancer-related deaths in American men [1]. Primary treatment includes androgen deprivation therapy, radiation, or radical prostatectomy [2], and despite recent advances in systemic and local treatment, for patients with metastatic disease survival outcomes remain poor [3, 4]. Moreover, the economic effects of this disease on the U.S. healthcare system are considerable with some authors estimating total expenditure at over 9.8 billion dollars in 2006 [5]. Recent data by Trogdon et al. revealed a 1.2 billion dollar cost for elderly men with prostate cancer in only 3 years [6]. Significant effort has been directed at identifying causes, complications, and risk factors for prostate cancer progression in order to improve survival and decrease the cost burden. However, there may be patient comorbidities that play a role in promoting disease progression and have yet to be identified [7, 8].

Recent translational research described a novel pathway that stimulated prostate cancer progression *in vivo* and *in vitro* including Cartilage Oligomeric Matrix Protein (COMP); a small (54 kD) molecule most commonly found in the extracellular matrix (ECM) of cartilage [9, 10]. Englund et al. described that COMP promoted progression by stimulating tumor invasion due to changes in intracellular calcium release from the endoplasmic reticulum and in oxidative phosphorylation [9]. The authors demonstrated the presence of COMP in 16% of tumor tissue microarrays and found a significant association with COMP expression and time to metastases and to biochemical recurrence (defined as a PSA > 0.2 ng/ml).

COMP also functions as an ECM stabilizer, and is upregulated with joint loading during exercise and secreted to the serum in patients with osteoarthritis [11]. However, the effects of COMP on prostate cancer progression in patients with osteoarthritis have not been studied and could present a novel therapeutic approach to prostate cancer treatment. The purpose of this study was to evaluate whether osteoarthritis was an independent risk factor for adverse outcomes of prostate cancer. Our secondary aim was to determine whether a diagnosis of osteoarthritis correlated with upregulation of tumor markers associated with angiogenesis (CD31), proliferation (Ki-67) and the presence of COMP. The study hypothesized that osteoarthritis is an independent risk factor for worse outcomes of prostate cancer patients as it pertains to overall survival, local recurrence, and metastasis while accounting for other comorbidities [9, 12]. The findings of this study could lead to a change in the standard of care for patients with osteoarthritis and prostate cancer and the potential value that orthopedic surgery could provide for these patients.

## RESULTS

A total of 274 patients with a mean age at prostate cancer diagnosis of 77.1 years (SD 9.33) were included in the study. The overall mean follow-up was 79.5 months (range 6.6 – 201.26). Survival of the entire cohort was 77% at last follow up. There were 78 patients (28%) with a concomitant diagnosis of osteoarthritis and 196 patients (72%) without osteoarthritis. Table 1 highlights the characteristics of the studied population as stratified by osteoarthritis presence. Of the 78 patients with osteoarthritis, 10.2% underwent joint arthroplasty.

**Table 1:** Demographic and patient characteristics based on osteoarthritis

Demographic Data Stratified by OA	Osteoarthritis (n=78)	No Osteoarthritis (n=196)	p value
Mean Age in Years at Diagnosis (SD)	66.9 (7.9)	65.9 (8.3)	0.383
Mean Follow Up Months Median (IQR)	60.8 (87.99)	85.3 (107.4)	0.332
Gleason Score (%)			
6 or below	26.9	44.9	0.016
7	35.9	32.7	
8 or higher	34.6	21.9	
Radiotherapy	100%	100%	1
Chemotherapy	7.70%	4.10%	0.064
Hormonal Therapy	58.5%	41.5%	0.015
Treatment with Surgery	11.70%	9.70%	0.531

There were no differences in patient demographics between the osteoarthritis and no osteoarthritis groups (Table 2). However, patients with osteoarthritis had a significantly higher Gleason score at the time of diagnosis compared to patients without osteoarthritis (Table 2). While overall survival was similar between groups, patients with osteoarthritis did had a significantly higher rate of metastatic disease during follow up compared to those without OA, 3% vs 1.8% (p=0.003).

**Table 2:** Clinical Outcomes

Outcomes of Prostate Cancer	Osteoarthritis (n=78)	No Osteoarthritis (n=196)	p value
Overall Survival (SD) in Months	81.6 (53.9)	89.1 (114.7)	0.600
Local Recurrence	1.50%	0.40%	0.878
Metastasis	3%	1.80%	0.003

### Osteoarthritis effects on mortality

In the logistic regression model evaluating independent predictors of mortality, only age at diagnosis (p=0.004) and use of chemotherapy (p<0.001) were independent predictors of death (Table 3). Survival time was similar with and without osteoarthritis while accounting for covariates (p=0.915). However, there was a numerical improvement in survival in OA patients who received joint arthroplasty that did not reach statistical significance (p=0.064).

**Table 3:** Logistic regression model predictive of mortality

Risk Factor	Odds Ratio	95% Confidence Interval		p value
Osteoarthritis	0.815	0.405	1.641	0.567
Age at diagnosis	1.059	1.018	1.100	*0.004*
Gleason Score	1.004	0.717	1.407	0.981
Chemotherapy	13.485	3.610	50.376	* <0.001*
Hormone Therapy	0.539	0.242	1.201	0.131
Prostatectomy	0.731	0.241	2.222	0.581
Constant	0.007			.002

### Osteoarthritis effects on local recurrence

The model used to identify independent predictors of local recurrence was statistically significant (X^2^: 21.53, p=0.006) at predicting local recurrence rates. Although this model correctly classified 98.5% of cases of local recurrence, osteoarthritis was not found to be an independent predictor of local recurrence as well as any other predictor included (p > 0.05).

### Osteoarthritis effects on distant metastasis

The model used to identify independent predictors of metastasis was statistically significant (X^2^: 47.04, p < 0.001) as it correctly classified 95.5% of patients who developed metastatic disease. There were four independent predictors of distant metastasis: age at diagnosis, surgical treatment, and a diagnosis of osteoarthritis (p<0.05). Table 4 demonstrates the odds ratios of each of the predictors. We then developed a model with the same predictors including joint arthroplasty, and chemotherapy and demonstrated similar statistical ability to discriminate metastasis (X^2^: 45.47, p<0.001) classifying 95.1 % of cases correctly. Notably, this model identified similar predictors of distant recurrence (age at diagnosis, treatment with chemotherapy, and treatment with surgery) except for a diagnosis of osteoarthritis (p=0.071) as demonstrated in Table 5.

**Table 4:** Logistic regression model predictive of metastasis

Risk Factor	Odds Ratio	95% Confidence Interval		p value
Osteoarthritis	5.244	1.493	18.414	*0.01*
Age at diagnosis	0.882	0.811	0.958	*0.003*
Gleason Score	0.261	0.178	1.172	0.103
Prostatectomy	4.557	1.144	18.160	*0.032*
Local Recurrence	10.057	0.351	288.349	0.178
Constant	.4708			0.630

**Table 5:** Modified Logistic regression model predictive of metastasis

Risk Factor	Odds Ratio	95% Confidence Interval		p value
Osteoarthritis	4.213	0.885	20.062	0.071
Age at diagnosis	0.897	0.821	0.980	*0.016*
Gleason Score	1.057	0.540	2.070	0.872
Chemotherapy Given	36.390	5.637	234.923	*0.000*
Prostatectomy	4.905	1.060	22.690	*0.042*
Joint Arthroplasty	2.792	0.457	17.042	0.266
Constant	6.214			0.610

### Histologic evaluation of prostate cancer samples

**Figure 1:**
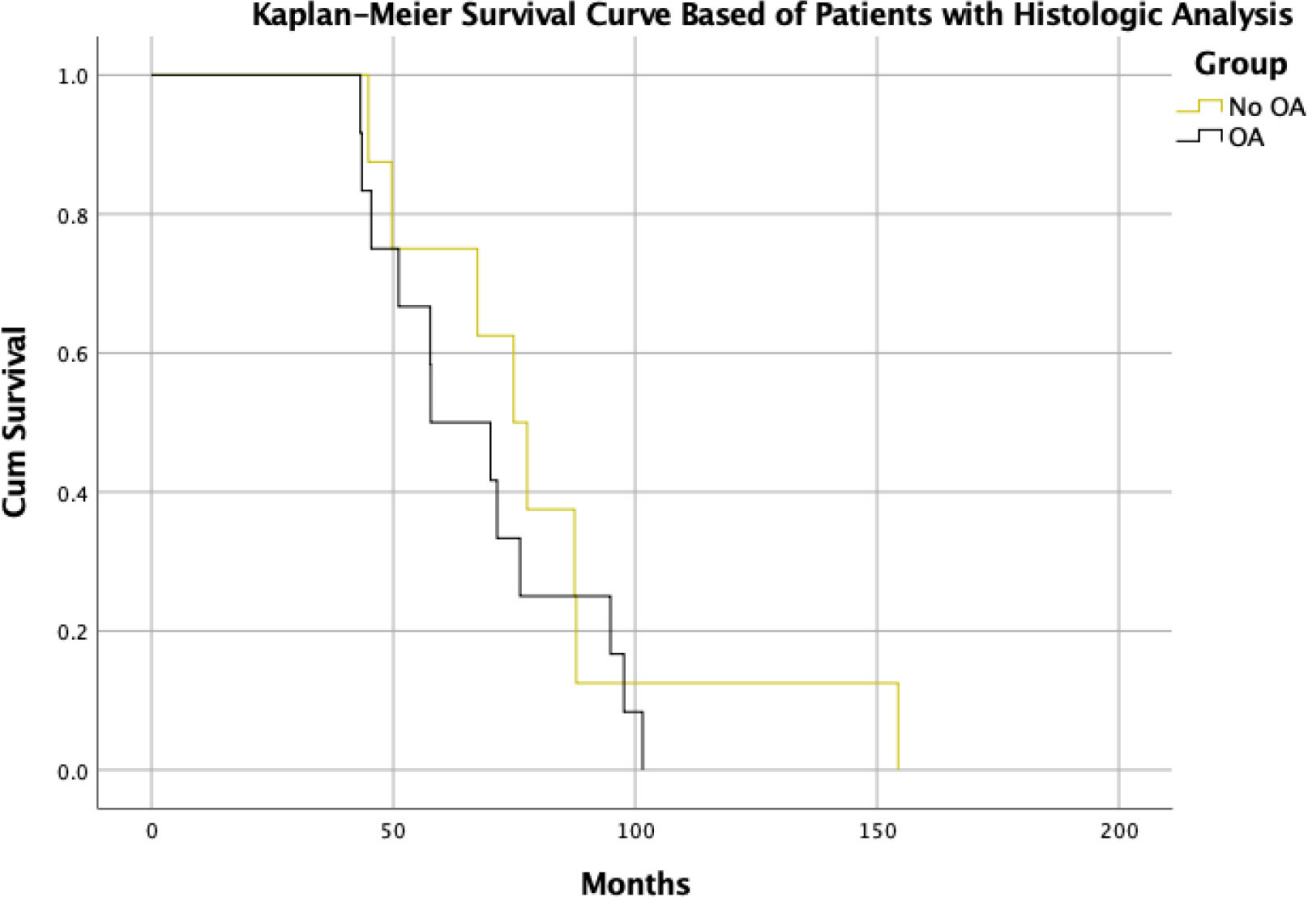
Kaplan-Meier Survival curve of patients with clinical and histological data.

**Figure 2:**
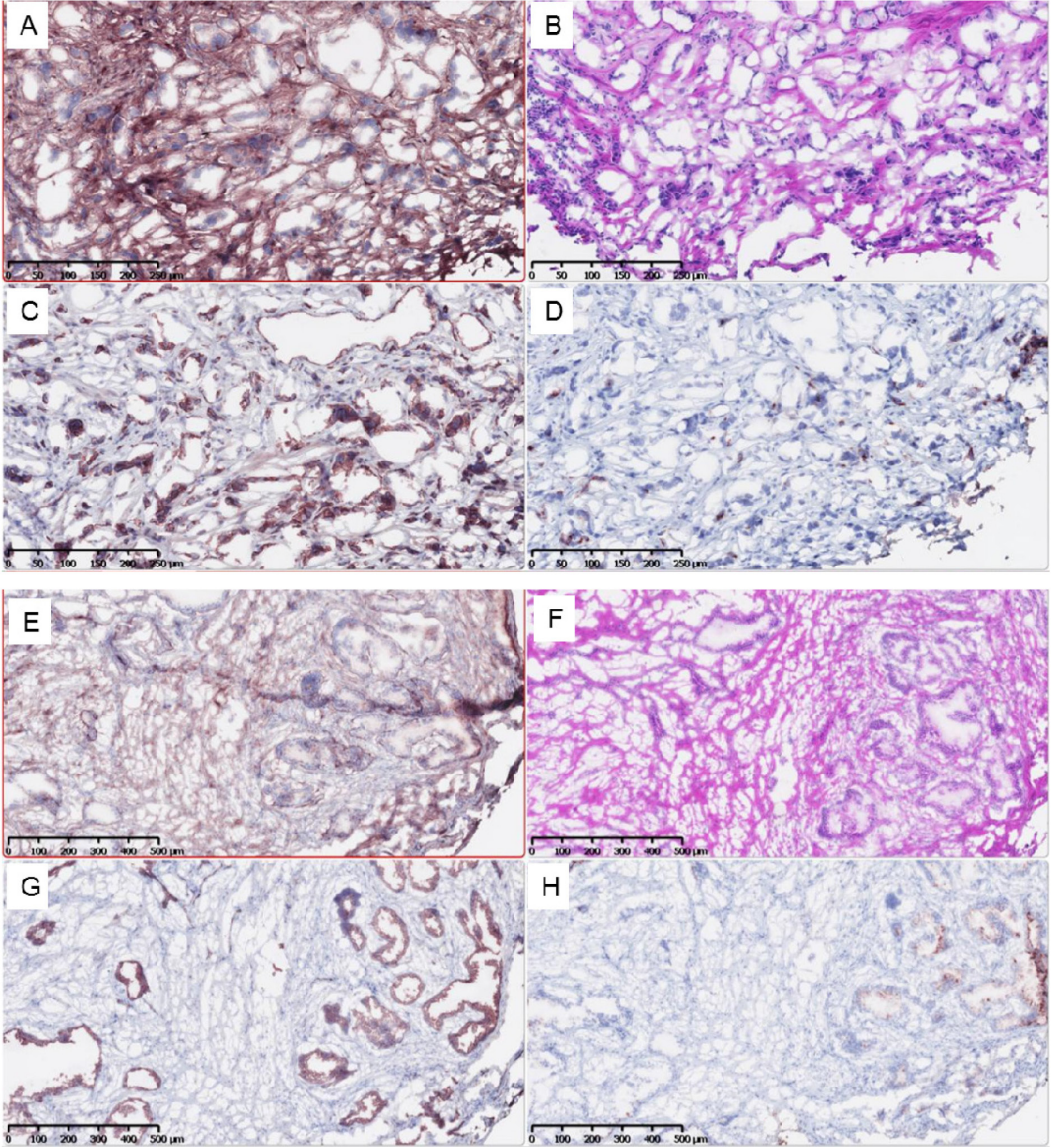
Lymph Nodes of Patient with osteoarthritis (top 4 images) and no osteoarthritis (bottom). **(A,E)** PSMA; **(B,F)** H&E, **(C,G)** Ki-67, **(D,H)** COMP

A total of twenty samples were evaluated histologically in this study; eight were of patients with osteoarthritis and twelve of those without osteoarthritis (Table 6, Figure 1 and 2). A total of seven samples from lymphatic nodes were available for analysis. In the prostate samples, COMP expression was significantly greater in patients with osteoarthritis (mean 23.9% vs 5.85%, p<0.05). The percent of positivity for CD31, Ki-67, and PSMA was similar in both groups (p>0.05 for all). Table 6 demonstrates the IHC depiction of the samples and the characteristics of the patients studied. Due to the limited sample size available, only one was of a patient with osteoarthritis, which coincided with the patient who expressed the greatest amount of COMP (61.3%), compared to 30.7% in the highest sample of patients without osteoarthritis (Table 7). The patient with osteoarthritis also presented with the highest Gleason score and developed metastatic disease (Figure 2).

**Table 6:** Immunohistochemistry Results and Patient Demographics of Prostate Samples

Patient	Age at diagnosis	OA	Survival (months)	Local Recurrence	Metastasis	Gleason Score	Initial PSA (ng/mL)	CD31	KI-67	COMP	PSMA	Surgery
1	71	-	154.38	No	No	3+4	n/a	0.8%	31.4%	80.3%*	2.8%	
2	77	-	87.5	No	No	4+5	27.9	3.1%	38.7%	12.2%	34.3%	
3	78	-	67.4	No	No	4+3	4.9	0.4%	0.6%	0.7%	3.1%	
4	74	-	74.9	No	No	4+4	9.4	0.2%	1.4%	0.7%	3.6%	
5	78	-	87.8	No	No	4+4	5.5	0.6%	2.4%	2.3%	17.1%	
6	60	-	49.7	No	No	3+4	4.7	0.6%	1.0%	0.1%	10.3%	
7	58	-	77.66	No	No	5+4	11	0.4%	3.8%	7.3%	4.3%	
8	69	-	44.84	No	No	4+3	11.28	0.6%	12.7%	17.6%	25.2%	
1	53	+	70.1	No	**Yes**	5+4	15	1.0%	3.8%	1.9%	12.4%	
2	72	+	101.55	No	No	3+4	6.98	0.7%	33.4%	37.0%	4.1%	
3	70	+	97.7	No	No	4+4	91	2.6%	72.7%	21.1%	39.2%	
4	74	+	76.22	No	No	3+5	258.1	1.1%	23.5%	8.8%	27.8%	
5	55	+	71.45	No	**Yes**	4+5	14.6	0.8%	0.8%	8.7%	24.7%	
6	65	+	45.43	No	No	3+4	8.6	0.6%	1.5%	4.9%	15.4%	THA, TKA
7	75	+	57.76	No	No	4+3	6.51	1.7%	18.1%	31.7%	28.1%	
8	61	+	57.66	No	No	4+5	22.3	0.5%	21.1%	35.0%	6.2%	
9	74	+	51.12	No	No	5+4	13.01	1.4%	40.6%	51.3%	5.2%	Bi-TKA, THA
10	60	+	43.22	No	No	4+3	5.76	0.3%	1.3%	37.0%	15.7%	TKA
11	70	+	43.49	No	No	4+5	67.38	0.7%	27.6%	47.2%	2.0%	Bi-THA
12	70	+	94.9	No	No	4+3	4.2	0.8%	1.7%	2.0%	2.4%	TKA

* Sample was excluded from analysis because of extreme outlier (>4SD from mean). OA: osteoarthritis; TKA: total knee arthroplasty. THA: total hip arthroplasty. Bi: bilateral.

**Table 7:** Immunohistochemistry Results and Patient Demographics of Lymph Node Samples

Patient	Age	Status	OA	% Positive PSMA	% Positive COMP	% Positive KI-67	Gleason Score	Initial PSA (ng/mL)	Local recurrence	Metastasis
1	74	alive	-	0.27%	7.06%	2.79%	3+3	4.48	0	0
2	76	alive	-	0.45%	27.08%	13.43%	3+3	6.1	0	0
4	81	alive	-	1.80%	30.71%	2.63%	4+4	17.5	0	0
5	65	alive	+	0.67%	61.39%	7.59%	5+4	15	0	1
6	71	alive	-	2.38%	16.35%	3.63%	stage 3	n/a	1	1
7	73	dead	-	7.48%	22.60%	7.84%	stage 4	n/a	1	1

OA: osteoarthritis

## DISCUSSION

The current study evaluated the influence of osteoarthritis and subsequent upregulation of COMP on the prostate cancer presentation and disease progression. Osteoarthritis was identified as an independent predictor of metastatic disease (OR 5.24, 95% CI 1.49 – 18.41, p=0.01). While the underlying pathophysiology remains to be elucidated, there may be similarities to rheumatoid arthritis [13, 14]. Although rheumatoid arthritis has a different pathophysiology from osteoarthritis, it leads to chronic joint damage that can stimulate COMP production and secretion into the blood stream [15]. Theoretically, joint injury leads to increased systemic COMP, which then stimulates tumor progression [15]. A registry based study of Medicare patients in Texas, correlated the presence of rheumatoid arthritis with increased mortality in prostate cancer patients by 50% after controlling for other comorbidities [14]. A more recent systematic review failed to identify rheumatoid arthritis as causing a greater risk of developing prostate cancer compared to the normal population [16] and these studies highlight the lack of knowledge on this topic. Our findings lead us to believe that osteoarthritis or rheumatoid arthritis could increase cancer proliferation but not cause tumor initiation, as has been demonstrated *in vitro* [9].

Patient survival was not influenced by osteoarthritis in this study. However, this finding may be confounded due to various factors. First, the disease usually progresses slowly with metastases developing after 10 years, and prostate cancer is often not the cause of death for patients, as death from cardiovascular disease predominates [17]. Second, despite a follow-up duration of over 6 years in the present cohort, the indolent nature of the prostate cancer may limit the power to detect a difference in overall survival. It may be possible that with longer follow-up, osteoarthritis may be associated with survival. Nonetheless, the survival rate of the patients in this cohort corresponding to findings from previous studies [17–19].

Osteoarthritis was not found to be an independent predictor of local cancer recurrence. This finding was not surprising as the likelihood of local recurrence has been more commonly correlated with pre-treatment PSA levels, Gleason scores, and various imaging parameters [20–22]. Moreover, local recurrence may be identified by biochemical recurrence, symptomatic disease, and/or radiographic findings, making its definition in the literature somewhat variable and limiting the interpretation of this lack of independent predictor identification [10, 17, 19–21].

An interesting finding that requires further study is that osteoarthritis was no longer an independent predictor of metastasis in patients who had undergone joint arthroplasty. While the underlying mechanism remains to be identified, this finding may have occurred due to self-selection of healthier patients undergoing arthroplasty for osteoarthritis versus surgeons not offering arthroplasty to patients with a high likelihood of unsatisfactory results (such as patients with advanced prostate cancer). Joint arthroplasty removes the areas of the joint involved in osteoarthritis and could thereby lead to decreased systemic levels of COMP, which in turn would reduce COMP-mediated promotion of cancer proliferation compared to osteoarthritis patients who did not undergo arthroplasty. The trend toward increased survival time of prostate cancer patients with osteoarthritis after joint arthroplasty warrants further investigation.

The IHC analysis revealed that COMP levels were significantly higher among biopsies of patients with osteoarthritis, compared to patients without osteoarthritis. This finding may be due to dysregulation of the ECM, promoted by increased COMP in the serum that travels systemically which could promote greater metastatic disease and distant seeding of tumor cells. Nonetheless, this is the first report of this relationship, and future studies are needed to evaluate systemic levels of COMP and possible temporal changes in COMP expression to further examine the interaction between osteoarthritis and prostate cancer.

Regarding the equal expression of CD31, PSMA and Ki-67 in both cohorts of patients, one could hypothesize that the greater metastatic disease seen in patients with osteoarthritis was not likely due to increased angiogenesis or direct tumor proliferation but due to cancer invasion into the adjacent tissues. Unfortunately, there are mixed opinions in the literature as for how these markers correlate with clinical tumor progression [23–25].

The observation of increased COMP expression in a lymph node of an osteoarthritis patient of twice the amount of that in a patient without osteoarthritis is promising, novel and the first of its kind in the literature. This finding could pertain to our hypothesis and is therefore currently being studied at our institution. However, we understand the limitations of our sample size and as such regard this as preliminary information.

Some limitations are inherent to our studies. The retrospective nature of this analysis may have increased the potential for selection bias not only due to the fact that our institution is a tertiary referral center but because of exclusion of patients without possible predictors and/or lack of data on them. Moreover, there is the possibility of confounding because the patients in this study received a comprehensive prostate cancer treatment protocol which included radiation therapy, chemotherapy, and surgical resection. Furthermore, inherent characteristics to the each of the compared groups may alter the findings of any regression model. As such, variables included in the model allowed us to account for initial differences, such as Gleason score and hormonal therarpy, as has been performed in the past [26–28]. Mean age at death was also not evaluated and the effects of osteoarthritis on patients who died at a young age not studied. We were unable to account for age at onset of osteoarthritis or age at which osteoarthritis was diagnosed, which may influence disease progression over time. An *a priori* power analysis was also not performed as no previous studies had evaluated the effect of osteoarthritis on prostate cancer and as such no estimation of an effect size could be made. The number of patients who underwent joint arthroplasty was limited; however, the relationship between arthroplasty and prostate cancer disease progression is novel and evaluations of larger patient cohorts are needed. Future studies will aim to determine whether high levels of COMP in the prostate or the serum and/or whether the severity of osteoarthritis impacts its relationship with prostate cancer.

In conclusion, this study identified a relationship between osteoarthritis and the development of metastatic disease in patients with prostate cancer. Patients with osteoarthritis expressed higher COMP levels in the prostate tissue and most likely in distant lymphatic nodes. Moreover, our findings suggest that joint arthroplasty may mitigate the effect of osteoarthritis on metastasis, which could impact treatment protocols and survival outcomes of the second most common cause of cancer-related deaths in men in the United States.

## MATERIALS AND METHODS

**Figure 3:**
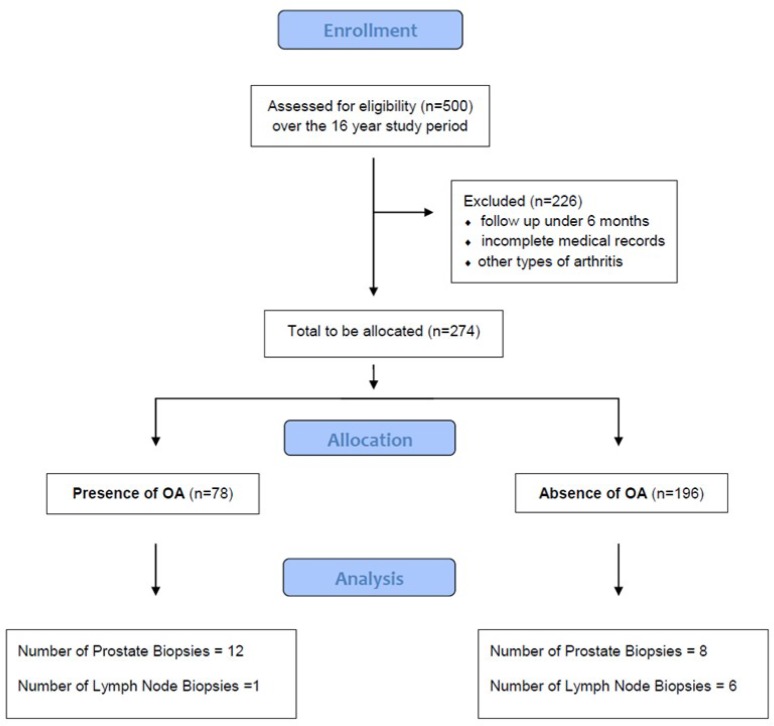
Consort Flow diagram of study design

A retrospective case-control study was performed by reviewing the clinical data of all prostate cancer patients treated at a tertiary referral center from 2000 to 2016. The study was approved by the Wake Forest University Health Sciences Institutional Review Board. Inclusion criteria comprised patients treated for prostate cancer at our institution over the age of 18, disease stage and follow up time over 6 months. Exclusion criteria were patients under the age of 18, and those with a tumor different from primary prostate cancer. Patients were stratified by concomitant diagnosis of osteoarthritis retrieved from the patients’ medical record and absence of osteoarthritis or joint diseases different from osteoarthritis (including rheumatoid arthritis) (Figure 3).

Data extracted included patient demographics, osteoarthritis presence or absence, Gleason score, history of joint surgery, type of joint surgery, use of radiation therapy, chemotherapy use, prostatectomy, hormonal therapy, age at diagnosis, progression free survival, and outcomes including local recurrence, distant metastasis, and death. Patient surgical history and imaging was assessed for total joint arthroplasty within the study period. Local recurrence was defined as clinical, radiographic or pathologic evidence of disease at the primary site or prostate bed. Distant metastasis was defined as any evidence of disease outside the primary and regional lymph nodal basins. Progression-free survival was defined as the duration of time to any disease recurrence or death. Time-to-event outcomes were calculated from the time of diagnosis to the event.

Immunohistochemistry (IHC) was used to evaluate prostate cancer samples and lymph nodes for a variety of markers with COMP being the main outcome of interest. Briefly, the cancer patient database was queried for tissue samples of patients included in this study. A total of 20 samples were identified and ordered at random by the Core Pathology Department at Wake Forest University. Full cohort evaluation was not possible due to the low availability of samples.

The prostate core biopsies and open biopsies were fixed with formalin and embedded in paraffin before sectioning. A total of four, 5μm slice samples were obtained for each of the 20 patients. The lymph nodes were obtained and prepared in a similar fashion. IHC protocols were performed following the manufacturer’s protocols. Hematoxylin and Eosin (H&E) staining was done as per our laboratories protocol. Pathology reports were extracted for the H&E stains to determine Gleason score per trained pathologists. IHC for angiogenesis using CD31 (Abcam, Cambridge, UK, Antibody ab28364 at 1:300 dilution with specificity for human, mouse and porcine tissue), COMP (Abcam, Cambridge, UK, Antibody ab231977 at 1:500 dilution with specificity for human and mouse samples), proliferation using Ki-67 (MilliporeSigma, Burlington, MA, Antibody ab9260 at 1:500 dilution with specificity for human, mouse and rat), and PSMA (prostate specific membrane antigen, Dako Agilent Technologies, Santa Clara, CA, m3620 at 1:70 dilution, reactive to human samples) were performed. A blinded reviewer calculated the percentage of positively stained cells by IHC within the tissues utilizing the VisioPharm Software (Hoersholm, Denmark) as previously described [29].

Statistical analysis was comprised of descriptive statistics, logistic regressions, and Cox regression models. The linearity of the continuous variables with respect to the logit of the dependent variable was assessed via the Box-Tidwell procedure. A Bonferroni correction was applied during assumption evaluation of the models. Initial unadjusted comparisons were also performed and presented herein. Model adjustments were made based on covariates including age at diagnosis, presence or absence of osteoarthritis, hormonal therapy, chemotherapy, Gleason score, and joint arthroplasty. Three different Cox models were performed to evaluate time to local recurrence, time to metastasis and time to death. Comparison of groups at baseline was done to test for possible differences and for determining if there was a need for propensity score matching. Chi-square tests and Mann-Whitney tests were used as appropriate following evaluation of data normality for the latter test. Given that the groups were clinically similar, as well as their treatment, it was deemed that propensity score matching was not necessary. Survival was estimated using the Kaplan-Meier method for the cohort of patients in which histology was performed. No statistical analysis was performed on the lymphatic node samples of IHC given the low sample size (n=7), as to avoid both type 1 and type 2 errors. On IHC analysis samples were excluded if their expression was 4 standard deviations above or below the mean (in total one sample was excluded).

## Abbreviations

COMP: Cartilage Oligomeric Matrix Protein
ECM: Extracellular Matrix (ECM)
PSMA: Prostate Specific Membrane Antigen
IHC: Immunohistochemistry
PSA: Prostate Specific Antigen
THA: Total Hip Arthroplasty
TKA: Total Knee Arthroplasty.
